# Metformin regulates multiple signaling pathways within castration-resistant human prostate cancer cells

**DOI:** 10.1186/s12885-022-10115-3

**Published:** 2022-09-29

**Authors:** Emuejevoke Olokpa, Sammed N. Mandape, Siddharth Pratap, La Monica V. Stewart

**Affiliations:** 1 Department of Biochemistry, Cancer Biology, Neuroscience and Pharmacology, Meharry Medical College, 1005 Dr. D.B. Todd Jr. Blvd., Nashville, TN 37208 USA; 2 School of Graduate Studies and Research, Bioinformatics Core, Meharry Medical College, 1005 Dr. D, B. Todd Jr. Blvd., Nashville, TN 37208 USA

**Keywords:** Prostate, Androgen receptor, p53, Metformin

## Abstract

**Background:**

The biguanide metformin has been shown to not only reduce circulating glucose levels but also suppress in vitro and in vivo growth of prostate cancer. However, the mechanisms underlying the anti-tumor effects of metformin in advanced prostate cancers are not fully understood. The goal of the present study was to define the signaling pathways regulated by metformin in androgen-receptor (AR) positive, castration-resistant prostate cancers.

**Methods:**

Our group used RNA sequencing (RNA-seq) to examine genes regulated by metformin within the C4–2 human prostate cancer cell line. Western blot analysis and quantitative RT-PCR were used to confirm alterations in gene expression and further explore regulation of protein expression by metformin.

**Results:**

Data from the RNA-seq analysis revealed that metformin alters the expression of genes products involved in metabolic pathways, the spliceosome, RNA transport, and protein processing within the endoplasmic reticulum. Gene products involved in ErbB, insulin, mTOR, TGF-β, MAPK, and Wnt signaling pathways are also regulated by metformin. A subset of metformin-regulated gene products were genes known to be direct transcriptional targets of p53 or AR. Western blot analyses and quantitative RT-PCR indicated these alterations in gene expression are due in part to metformin-induced reductions in AR mRNA and protein levels.

**Conclusions:**

Together, our results suggest metformin regulates multiple pathways linked to tumor growth and progression within advanced prostate cancer cells.

**Supplementary Information:**

The online version contains supplementary material available at 10.1186/s12885-022-10115-3.

## Background

The biguanide metformin (1, 1- dimethylbiguanide hydrochloride) is currently one of the most commonly prescribed oral medications for the treatment of type II diabetes in the United States. Approved by the Federal Drug Administration in 1994, metformin is believed to reduce serum glucose levels by reducing gluconeogenesis and glycogenolysis in the liver and producing additional effects within the intestinal tract (reviewed in [1]). In addition to its antidiabetic effects, metformin has been shown to improve other disease states. Metformin has been used clinically to regulate the ovary and correct endocrine dysfunction in women suffering from polycystic ovary disease (PCOS) [2]. There is evidence metformin reduces cognitive impairment and may serve as an effective treatment for neurological disorders such as depression, Alzheimer’s disease, and Parkinson’s Disorder [3, 4]. Metformin exposure can reduce acute kidney injury and other types of renal damage [5, 6]. Furthermore, multiple studies have shown that metformin can suppress the growth and progression of prostate cancer and other types of carcinomas. Data from in vitro studies have shown that metformin exposure reduces proliferation and invasion of human prostate cancer cell lines [7–11], while in vivo studies have revealed metformin reduces prostate tumor growth in xenograft and transgenic mouse models [8, 9]. Epidemiological studies also indicate that metformin may reduce prostate tumor development and progression. A retrospective study by D.E. Spratt et al. suggests that metformin reduces the risk of PSA recurrence and distant metastases in prostate cancer patients undergoing radiotherapy as well as the development of castration-resistant prostate cancer [12]. Metformin exposure also reduced biochemical recurrence and increased overall survival in patients with localized prostate cancer treated with radiation therapy [13]. Improved overall survival was noted in diabetic prostate cancer patients taking metformin in a retrospective study of patients with advanced prostate cancer performed by J.A. Richards et al. [14]. A case-control study from J.L. Wright and J.L. Stanford also noted a decreased relative risk of prostate cancer in Caucasian men taking metformin [15]. However, not all studies have detected a change in prostate cancer risk following metformin exposure. For example, in a more extensive case-control study performed by L. Azoulay et al., no reduction in prostate cancer risk was noted in patients with type 2 diabetes who took metformin [16]. Therefore, metformin may suppress prostate tumor growth only in a subset of prostate cancer patients.

While preclinical studies have shown metformin suppresses the growth of prostate tumors, the mechanism by which metformin produces anti-tumor effects is not fully understood. Multiple studies have demonstrated that metformin exposure inhibits cell cycle progression by inducing G_0_/G_1_ arrest within human prostate cancer cells [8, 9, 17–19]. The tumor suppressor p53 is known to regulate cell cycle progression by modulating the expression of proteins such as the cyclin dependent kinase inhibitor p21, 14–3-3-σ and GADD45α [20]. p53 appears to play a critical role in metformin-induced responses, for the ability of metformin to inhibit proliferation is reduced in prostate cancer cells that do not express wild type p53 or where p53 expression has been knocked down via siRNA [18]. However, other signaling pathways may contribute to the anti-tumor effects of metformin. Concentrations of metformin that inhibit prostate cancer cell proliferation and tumor growth also lower the expression of the proto-oncogene c-Myc in a p53-independent manner [8]. It is, therefore, possible metformin suppresses prostate tumor growth by regulating the activity of additional pathways that control prostate cancer growth and survival.

It is also not clear whether metformin produces anti-tumor effects in all forms of advanced prostate cancers. Prostate cancer begins as a disease that requires androgens such as dihydrotestosterone (DHT) for growth. Following the administration of therapies that target androgen receptor (AR) signaling, prostate tumors progress to castration-resistant forms that are less dependent on circulating androgen levels. To date, most studies have examined the effects of metformin in the castration-sensitive LNCaP or the castration-resistant PC3 and DU145 human prostate cancer cell lines. The LNCaP cells, which express a mutant form of the AR [21, 22], are often used as a model of early-stage prostate cancers. Many human castration-resistant prostate tumors retain expression of AR. However, little to no AR is expressed within the PC3 and DU145 cells [21]. At present, our understanding of the extent to which metformin suppresses growth and intracellular signaling in AR-positive, castration-resistant prostate cancers is limited. The goal of the current study was to examine the signaling pathways regulated by metformin in AR positive, castration-resistant prostate cancers. Our data indicate that growth inhibitory concentrations of metformin regulate not only AR signaling but also other pathways critical for the survival of human prostate cancer cells.

## Methods

### Materials

DMEM low glucose media, DMEM/F-12 media (1:1), Hams’ F-12 media, RPMI 1640 media, penicillin/streptomycin solution, phosphate buffered saline (PBS), Pierce RIPA buffer, and the Presto Blue cell viability reagent were purchased from ThermoFisher Scientific (Waltham, MA). Metformin and the media additives d-biotin, adenine hemisulfate, insulin solution, and apo-transferrin were purchased from Sigma Aldrich (St. Louis, MO). Fetal bovine serum (FBS) was obtained from HyClone (Logan, UT). Zapoglobin and Isoton II were purchased from Beckman Coulter Inc. (Fullerton, CA). Rabbit anti-mouse IgG secondary antibody was obtained from Zymed Laboratories, Inc. Both horseradish peroxidase-conjugated donkey anti-rabbit and sheep anti-mouse antibodies were purchased from GE Healthcare Biosciences (Pittsburg, PA). All tissue culture plasticware and additional chemicals were purchased from ThermoFisher Scientific.

### Cell culture

The C4–2 cell line was purchased from ViroMed Laboratories (Burlington, North Carolina) and grown in T medium (80% DMEM low glucose medium, 20% Hams’ F12 medium, 5% heat inactivated FBS, 1% penicillin/streptomycin, 0.244 μg/ml d-biotin, 25 μg/ml adenine hemisulfate, 5 μg/ml insulin and 5 μg/ml apotransferrin). The C4–2B, 22Rv1 and PC-3 cell lines were purchased from American Type Culture Collection (ATCC, Manassas, VA). The C4–2B and 22Rv1 cell line were grown in RPMI 1640 media supplemented with 10% FBS and 1% penicillin/streptomycin. PC-3 cells were cultured in DMEM-F12 medium supplemented with 10% FBS and 1% penicillin/streptomycin. Each cell line was maintained in an incubator with a 5% CO_2_ atmosphere at 37 °C.

### Cell proliferation assays

For cell count assays, each cell line was plated in 6 well plates at a density of 10,000–20,000 cells per well and allowed to attach overnight. The cells were then treated for 4 days with PBS vehicle or metformin (0.2–20 mM). Following treatment, the cells were detached using trypsin-EDTA and counted using a Coulter counter. To measure cell viability, each cell line was first plated in 24 well plates at a density of 5000–10,000 cells/well. Cells were then treated for 3 days with PBS vehicle or metformin (1–10 mM). The cells were next incubated at 37 °C for 1–2 hours with the PrestoBlue cell viability reagent (100 μl PrestoBlue/ml culture media). The amount of relative fluorescence of the culture media was then measured at an excitation wavelength of 535 nm and emission wavelength of 615 nm.

### RNA-sequencing

C4–2 cells were plated in 10 cm dishes at a density of ~ 750,000 million cells/dish and allowed to attach for 2 days. The cells were then treated for 24 hours with either PBS vehicle or 5 mM metformin. Total RNA was extracted from treated cells using the Trizol reagent and further purified using the Qiagen RNeasy Mini kit. Each RNA sample (three replicates per treatment group) was validated for RNA integrity using an Agilent 2100 Bioanalyzer. RNA-sequencing (RNA-seq), including library preparation, was conducted at the Vanderbilt Technologies for Advanced Genomics (VANTAGE) core facility. A standardized TruSeq mRNA protocol was used for library preparation, followed by sequence runs on an Illumina HiSeq 2500 utilizing a 50-bp paired-end RNA-Seq protocol.

### Bioinformatics analysis

Bioinformatics analysis of the RNA-seq read data was performed at the Meharry Bioinformatics Core facility. Qiagen CLC Genomics Workbench Version 7.0 (Hilden, Germany) was used for mapping alignments to determine transcript expression levels by using FPKM methodology and produced a Differential Gene Expression (DGE) list. Biological network analysis for downstream pathway enrichment was conducted using WebGestalt [23, 24] to obtain significantly enriched Kyoto Encyclopedia of Genes and Genomes (KEGG) pathways and Gene Ontology (GO) Biological Process terms [25, 26]. Pathway visualizations and biological interaction network characterizations were performed using CYTOSCAPE bioinformatics platform with Michigan Molecular Interactions Database (MIMI) and GeneMANIA plugins [27–29]. The Venny program [http://www.stefanjol.nl/venny] was used to compare genes from our differential gene expression (DGE) list with AR target genes previously identified by C.E. Massie et al. [30] and p53 target genes identified by M. Fischer [31].

### qRT-PCR analysis

To determine the effect of metformin on mRNA expression, each cell line was plated in FBS-containing media and treated for 24 hours with PBS vehicle or millimolar concentrations of metformin. Total RNA was then isolated from treated cells using the Trizol reagent according to the manufacturer’s protocol. For each sample the iScript cDNA Synthesis Kit (BioRad, Hercules, California) was used to synthesize cDNA from 1 μg of total RNA. The cDNA was then amplified by quantitative PCR using a reaction involving iQ SYBR Green Supermix reagent (BioRad). This PCR reaction consisted of an initial denaturation step (3 min at 95 °C) and 40 cycles of PCR (95 °C for 30 s, 55 °C for 30 s and 72 °C for 30 s). Quantitect primer sets from Qiagen (Valencia, CA) were used to detect the presence of GR, Nxk3.1, PARP1 and PPARγ. The primer set used to detect the presence of full-length AR (AR-FL) was forward 5′-CAG CCT ATT GCG AGA GAG CTG-3′ and reverse 5′-GAA AGG ATC TTG GGC ACT TGC-3′. Primers used to detect 18S rRNA were forward 5′-ATC AAC TTT CGA TGG TAG TCG-3′ and reverse 5′-TCC TTG GAT GTG GTA GCG-3′. The ΔΔCt algorithm was used to calculate the relative RNA amounts. RNA levels for each gene product of interest were normalized to 18S rRNA levels.

### Western blot analysis

To measure the effect of metformin on AR-FL protein levels, C4–2 cells were plated at a density of 600,000–750,000 cells per 10 cm dish in T media supplemented with 5% heat inactivated FBS. The cells were then treated with PBS vehicle (EtOH) or the indicated concentrations of metformin for 24 hours. Following drug exposure, cells were lysed using RIPA buffer (Thermo Scientific, Pittsburg, PA) to prepare whole cell extracts. Protein concentrations for each sample were calculated using the Bradford protein assay (BioRad, Hercules, CA). Equal amounts of protein from each extract were separated on SDS-PAGE gels and transferred to a nitrocellulose membrane. Membrane blots were then blocked in TBST (1x TBS, 0.1% Tween 20) containing 1% non-fat powdered milk and incubated with AR mouse monoclonal antibody (clone AR 441, Lab Vision Corporation, Fremont, CA; 1:400) diluted in the blocking solution overnight at 4 °C. The blots were next washed in blocking buffer and incubated with sheep anti-mouse secondary antibody conjugated to horseradish peroxidase. Proteins were then visualized using the Pierce Enzyme-Linked Chemiluminescence (ECL) Western Blotting Substrate (Thermo Scientific). ECL images were captured using either X-ray film or the Carestream Gel Logic 4000 imaging system. Blots were reprobed with an actin mouse monoclonal antibody (Chemicon International, Temecula, CA; 1:10,000) to confirm equal loading of the gel.

### Statistical analysis

Each experiment was performed at least three times, and representative data are shown. For proliferation assays and qRT-PCR experiments, One Way Analysis of Variance (ANOVA) analyses were performed using the Sigma Stat 3.1 (Systat Software Inc.) or GraphPad Prism 7 program (GraphPad Software) to detect differences between control and treatment groups. The standard for statistical significance was *P* < 0.05.

## Results

### Metformin inhibits proliferation of AR positive, castration-resistant prostate cancer cells

**Fig. 1:**
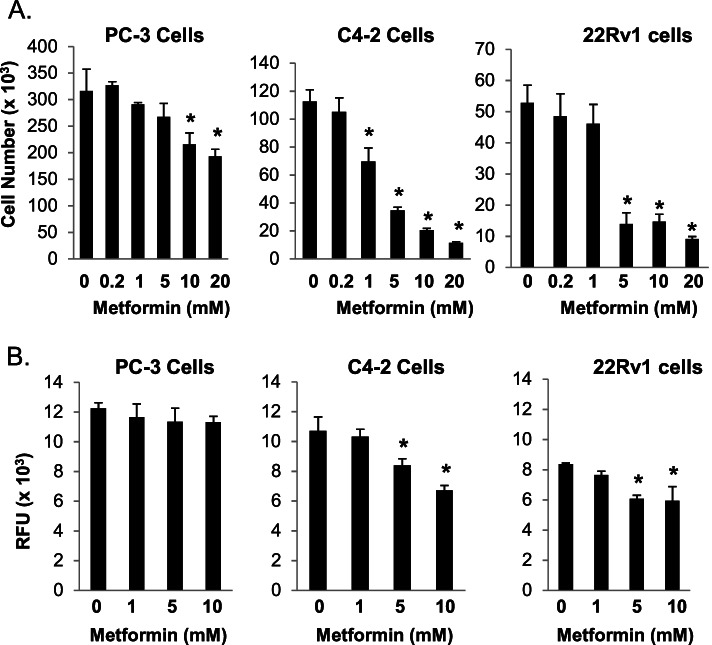
Metformin inhibits proliferation of castration-resistant C4–2, 22Rv1 and PC-3 prostate cancer cells. **A** Each cell line was treated with either PBS vehicle or different concentrations of metformin (0.2–20 mM) for 4 days. Following treatment, the cells were detached from the tissue culture plate and counted using a Coulter counter. Each bar represents the mean ± SD for three wells. *, *P* > 0.05 compared to PBS vehicle (0 mM metformin). **B** Cells were first treated with PBS vehicle or metformin (1–10 mM) for 4 days. The PrestoBlue assay was then performed to measure the viability of treated cells. Each bar represents the mean ± SD for four wells. *, *P* > 0.05 compared to phosphate buffered saline (PBS) vehicle (0 mM metformin)

Previous studies indicate that millimolar concentrations of metformin reduce proliferation of androgen receptor (AR) negative, castration-resistant human prostate cancer cell lines [9, 10]. To determine whether metformin alters proliferation of castration-resistant prostate cancer cells that express AR, we examined the effects of metformin on the AR-positive C4–2 and 22Rv1 human prostate cancer cells. As a control, we also tested metformin in PC-3 cells, a castration-resistant human prostate cancer cell line that expresses little to no AR. Cell count–based assays revealed that metformin concentrations ≥5 mM significantly reduced the proliferation of C4–2, 22Rv1 and PC-3 cells grown in FBS containing media (Fig. 1A). Furthermore, Presto Blue assays demonstrated that metformin exposure reduced viability of C4–2 and 22Rv1 cells (Fig. 1B). The results of the cell count and Presto Blue assays suggest the AR-positive C4–2 and 22Rv1 cells are more sensitive to the effects of metformin than the PC-3 cell line. Significant alterations in PC-3 cell proliferation required metformin concentrations ≥10 mM. However, lower metformin concentrations significantly reduced proliferation and viability in the C4–2 and 22Rv1 cell lines.

### Multiple gene pathways are regulated by metformin

**Fig. 2:**
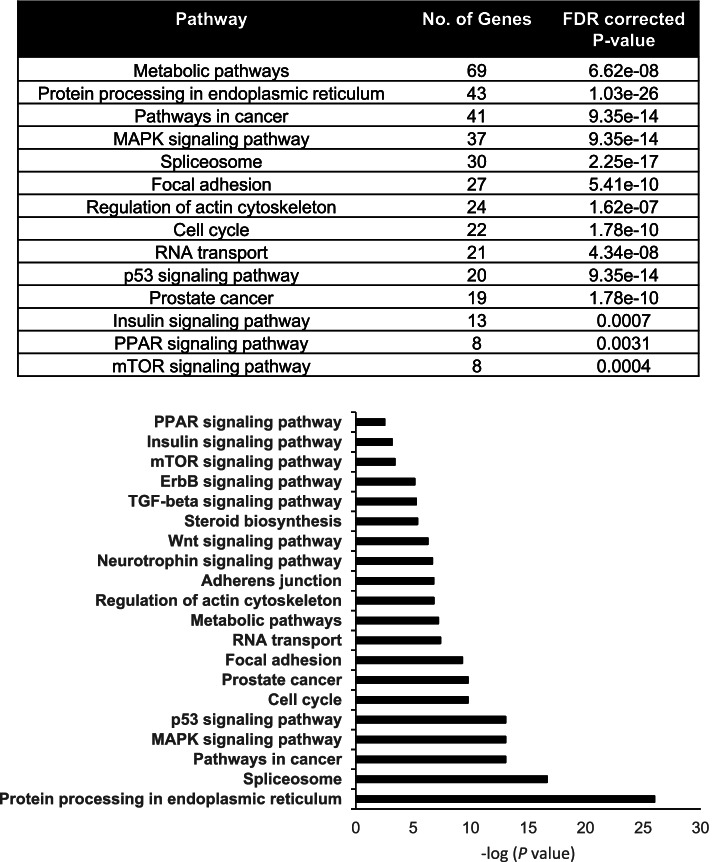
Metformin regulates multiple signaling pathways within C4–2 cells. RNA-sequencing was performed on total RNA extracted from C4–2 cells treated with phosphate buffered saline (PBS) vehicle or 5 mM metformin. Downstream pathway enrichment analysis of significantly changed transcripts was conducted using WebGestalt to obtain significantly enriched KEGG pathways and GO Biological Process terms. Pathway visualizations and biological interaction network characterizations were performed using the Cytoscape bioinformatics platform with the Michigan Molecular Interactions Database (MIMI) and GeneMANIA plugins

RNA-seq was performed to globally identify the gene products altered in C4–2 cells with metformin exposure. In this study, we compared RNA levels between cells treated with PBS vehicle and cells exposed to 5 mM metformin since this concentration significantly reduced the proliferation of C4–2 cells. Metformin exposure significantly altered the expression of 1298 genes within the C4–2 cell line. KEGG pathway and gene ontology analysis of the RNA-seq data demonstrated that metformin alters the expression of 69 gene products associated with metabolic pathways. However, this analysis also revealed that metformin regulates genes that play critical roles in the spliceosome, RNA transport, and protein processing within the endoplasmic reticulum (Fig. 2). Expression of genes that regulate focal adhesion, adherens junctions, and the actin cytoskeleton were also altered in C4–2 cells treated with metformin. Furthermore, metformin exposure modulated expression of genes involved in multiple pathways associated with cancer growth and progression (Fig. 2 and Table 1). Gene products associated with the ErbB, insulin, mTOR, TGF-β, MAPK, and Wnt signaling pathways were altered in cells exposed to metformin. Metformin regulated expression of 19 genes associated with prostate cancer, such as AR, Nxk3.1, KLK3 (PSA), CDKN1A (p21), CDKN1B (p27), and CCND1 (cyclin D1) (Table 1 and data not shown). It also increased expression of gene targets that contribute to p53 signaling, such as GADD45 (α, β, and γ), MDM2, SIAH-1, and thrombospondin 1. Within the PPAR signaling pathway, metformin exposure significantly increased expression of PPARγ, and reduced the level of carnitine palmitoyltransferase 2 (CPT2) mRNA. In addition to the changes noted above, metformin treatment produced a 1.83 fold increase in the lncRNA MALAT-1 and a 6.08 fold reduction in PARP1 expression (Table 1).

**Table 1:** Selected gene products regulated by metformin

Pathway	GENE ID	Fold Change	FDR Corrected ***P***-value
p53 Signaling	MDM2	2.43	2.44E-05
	SIAH-1	4.14	2.57E-02
	GADD45a	5.41	2.45E-10
	GADD45b	6.68	1.19E-03
	GADD45g	4.88	9.80E-09
	THBS1	4.87	3.59E-15
Prostate Cancer	PSA (KLK3)	−3.85	2.45E-104
	KLK2	−3.55	7.70E-33
	NKX3.1	−3.24	8.26E-102
	CDKN1A (p21)	4.86	1.28E-10
	CDKN1B (p27)	1.92	8.94E-03
	CCND1	−4.66	1.87E-08
	CREBBP	4.70	2.44E-10
PPAR Signaling	CPT2	−11.28	4.76E-06
	UBC	2.83	3.51E-11
	PPARG	3.91	1.16E-03
Other Cancer Related	MYC	−2.91	1.07E-11
	PARP1	−6.08	4.75E-16
	DUSP1	225.85	6.87E-102
	JUN	22.22	1.34E-146
	FAS	12.24	8.83E-06
	HES1	5.79	7.44E-06
	MALAT1	1.83	9.07E-12

**Fig. 3:**
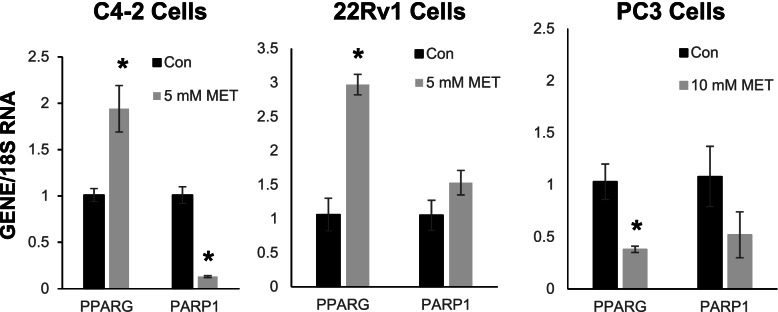
Metformin induces expression of PPARγ in C4–2 and 22Rv1 but not PC-3 prostate cancer cells. C4-2, 22Rv1 and PC-3 cells were treated for 24 hours with either phosphate buffered saline (PBS) vehicle or metformin (5–10 mM). Total RNA was then extracted from treated cells. qRT-PCR was performed to measure the amount of PARP1, PPARγ mRNA and 18S rRNA in each sample. The ΔΔCT method was used to calculate the relative amount of PARP1 and PPARG in treated cells. Each bar represents the mean −/+ SEM for three independent samples. *, *P* < 0.05 compared to PBS control

We confirmed by qRT-PCR that PPARγ mRNA levels were increased and PARP1 mRNA expression was reduced in C4–2 cells exposed to 5 mM metformin (Fig. 3). We also examined the regulation of these gene products in 22Rv1 and PC-3 cells. Metformin (5 mM) significantly increased PPARγ mRNA within 22Rv1 cells. However, it did not lower PARP1 expression within the 22Rv1 cell line. In PC3 cells, which were less responsive to the anti-proliferative effects of metformin, treatment with metformin did not significantly lower PARP1 or induce PPARγ mRNA levels.

### Metformin regulates a subset of AR target genes

**Fig. 4:**
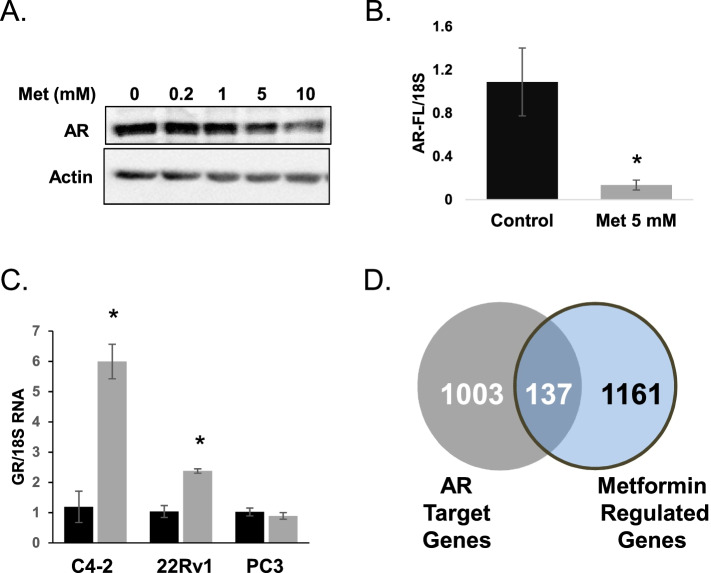
Metformin regulates androgen receptor (AR) signaling and a subset of AR target genes within C4–2 cells. **A** C4–2 cells were treated for 24 hours with phosphate buffered saline (PBS) vehicle or metformin (0.2–10 mM). Western blot analysis was performed to determine AR and actin protein levels in treated cells. **B** C4–2 cells were treated with PBS vehicle or 5 mM metformin for 24 hours. qRT-PCR was performed on total RNA extracted from treated cells to measure the level of full-length AR (AR-FL) and 18S RNA. Each bar represents the mean −/+ SEM for three independent samples. *, *P* < 0.05 compared to PBS control. **C** qRT-PCR was performed to detect the level of GR and 18S RNA in C4–2 and 22Rv1 cells grown in FBS-containing media and exposed to PBS vehicle or 5 mM metformin. Each bar represents the mean −/+ SEM for three independent samples. *, *P* < 0.05 compared to PBS control. **D** Venny program was used to compare the list of metformin regulated genes identified by RNA-Seq to the AR direct target genes identified by C. Massie [30]

RNA-seq analysis revealed that many of the prostate cancer related gene products regulated by metformin within the C4–2 cell line are also AR target genes. Metformin lowered expression of gene products that are induced by AR signaling, such as kallikrein 2, PSA (kallikrein 3), Nkx3.1, and the DNA repair gene PARP1. Furthermore, metformin exposure increased mRNA levels of PPARγ, a nuclear receptor whose expression is suppressed by activation of AR [32]. We therefore hypothesized that the majority of alterations in gene expression detected by RNA-seq in the C4–2 cells were due to metformin-driven alterations in AR signaling. To test this hypothesis, we first examined the effect of metformin on AR protein expression. Millimolar concentrations of metformin lowered protein levels of full-length AR (AR-FL) within the C4–2 cell line (Fig. 4A). In addition, 5 mM metformin reduced the presence of AR-FL mRNA within C4–2 cells (Fig. 4B). We next examined the ability of metformin to regulate additional proteins regulated by AR signaling. Previous studies have noted AR activation suppresses the expression of the glucocorticoid receptor (GR) in prostate cancers [33]. In both C4–2 and 22Rv1 cells, micromolar concentrations of metformin increased GR mRNA levels (Fig. 4C). Metformin also suppressed the ability of DHT to upregulate Nkx3.1 and downregulate GR in C4–2B cells (data not shown). To determine further the extent to which metformin regulates AR target genes within C4–2 cells, we compared the list of metformin-regulated genes obtained from our RNA-seq experiment with the list of AR target genes identified by C. Massie et al. via ChIP-seq analysis [30]. Approximately 10% of the gene products identified in our analysis were characterized as direct AR genes by the Massie et al. group. However, most of the gene products detected by RNA-seq appear to be regulated independently of AR (Fig. 4D).

### Metformin regulates a subset of p53 target genes within C4–2 cells

**Fig. 5:**
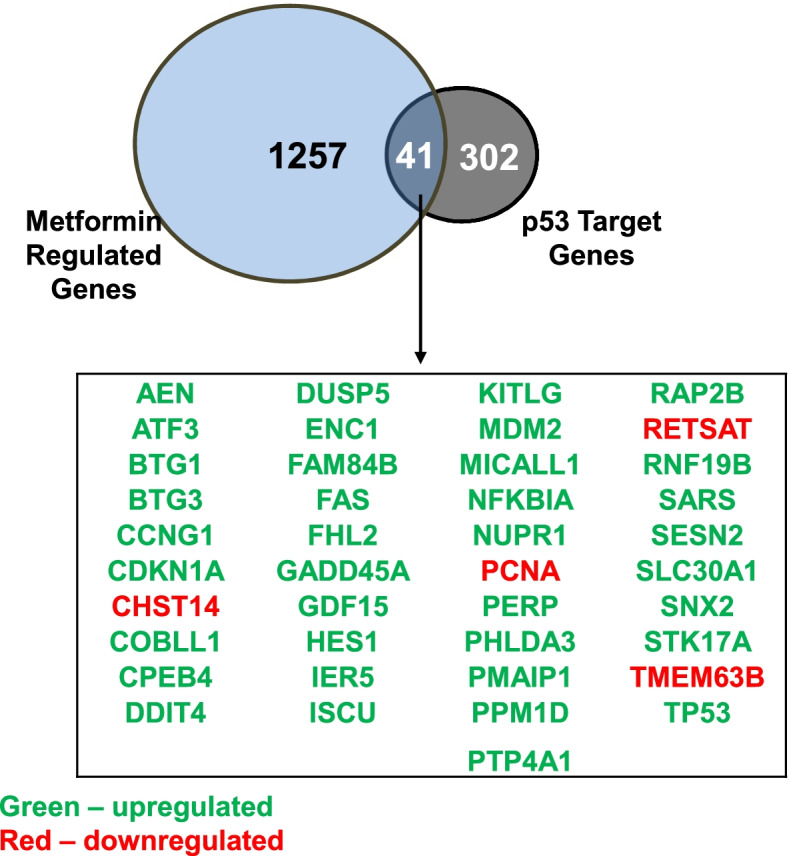
Metformin alters expression of p53 target genes within C4–2 cells. RNA-sequencing was initially performed to identify metformin-regulated genes in the C4–2 cell line. The list of metformin-regulated genes identified by this analysis was then compared to p53 direct target genes characterized by M Fischer et al. [31] using the Venny program. The box inset lists the 41 p53 target genes that are also regulated by metformin

Some studies have proposed that the tumor suppressor p53 contributes to metformin-induced responses in prostate cancer. Work by I. Ben Sahra and colleagues demonstrated that metformin induces expression of REDD1 (also known as DDIT4), a negative regulator of mTOR, and inhibits proliferation of human prostate cancer cell lines in a p53-dependent manner [18]. Furthermore, the presence of p53 enhances the ability of combination treatments involving metformin to induce apoptosis in prostate cancer cells [34, 35]. Unlike the PC-3 cells, the C4–2 cell line expresses wild-type p53 protein [36]. It is therefore possible that the metformin-mediated alterations in gene expression we noted in C4–2 cells require p53. To determine what percentage of metformin-regulated genes were also p53 target genes, we compared a list of 343 genes identified as direct targets of p53 by M. Fischer et al. [31] to the metformin regulated genes detected by RNA-seq. This analysis revealed that less than 4% of metformin regulated gene targets (41 gene targets) were also p53 direct targets. Metformin exposure induced expression of TP53, the gene that encodes p53, as well as p53 target genes associated with cell cycle and apoptosis, such as GADD45A, CDKN1A, DDIT4, FAS, and MDM2. Conversely, metformin reduced expression of the cell proliferation marker PCNA, two genes that encode metabolic enzymes (RETSAT and CHST14), and the gene for the cation channel TMEM63B (Fig. 5).

## Discussion

Metformin is an antidiabetic agent that not only controls serum glucose levels but also suppresses the growth of prostate cancers. However, our limited knowledge of the mechanism underlying the anti-tumor effects of metformin makes it difficult to identify patients that could benefit from therapeutic regimens involving this drug. To enhance our understanding, our group used RNA-seq to characterize the molecular changes induced by metformin in AR-positive, castration-resistant prostate cancer cells. The data obtained from this study revealed that metformin regulates multiple signaling pathways that contribute to cancer cell growth. As expected, metformin alters the expression of genes involved in metabolism. It also modulates genes involved in RNA transport and protein processing. In addition, metformin regulates the expression of AR target genes as well as genes that play critical roles in other growth factor signaling pathways. To our knowledge, this is the first report that has examined global changes in gene expression induced by metformin within AR-positive prostate cancer cells.

AR is a key driver of early and late-stage prostate cancers. Early stage prostate cancers primarily express the full-length, 110 kDa version of the AR (also known as AR-FL), while AR-V7 and other AR variants have been detected within advanced prostate cancers and tumors that have developed resistance to androgen-deprivation therapy or antiandrogens [37]. Cell proliferation studies suggested that AR levels might influence the ability of prostate cancer cells to respond to metformin, for the AR-negative PC-3 cells were less sensitive to the anti-tumor effects of metformin than the AR-positive C4–2 and 22Rv1 cells. This was supported by data from the RNA-seq study, which demonstrated metformin regulates multiple AR target genes within C4–2 cells. Alterations in the level of AR target genes we noted within C4–2 cells are likely due in part to metformin-driven decreases in AR function, for metformin reduced AR-FL protein and mRNA levels. Our data agree with work by J. Wang et al., who demonstrated millimolar levels of metformin lower protein levels of AR-FL in LNCaP cells and AR-FL as well as AR-V7 in the CWR22Rv1 cell line [38]. Since metformin suppresses AR expression within advanced prostate cancer cells, it may serve as an effective way to control AR signaling in drug-resistant cancers. While there was some overlap between genes regulated by metformin and AR target genes, not all genes regulated by metformin are direct transcriptional targets of AR. This suggests that metformin may use both AR-dependent and AR-independent pathways to regulate prostate cancer proliferation.

AR is not the only member of the nuclear receptor superfamily whose expression was altered by metformin. Our study indicates that metformin exposure induces the expression of at least two additional nuclear receptors, GR and PPARγ. We believe that this is due in part to metformin-induced decreases in AR activity, for expression of PPARγ and GR is suppressed by AR in human prostate cancer cells [32, 33]. GR appears to be a key regulator of advanced prostate cancers, for GR expression is elevated in metastatic prostate cancers, particularly those that have developed resistance to drugs that target the AR signaling pathway [39, 40]. Reductions in GR function inhibit prostate cancer cell proliferation and increase sensitivity to abiraterone [40]. The role of PPARγ in prostate cancer is somewhat more complicated. PPARγ can function as a tumor suppressor, for loss of PPARγ within the mouse prostate results in the development of prostate intraepithelial neoplasia (PIN), a precursor to prostate cancer [41]. PPARγ agonists have also been shown to reduce prostate cancer growth in vitro and in vivo [42–47]. However, work performed by I. Ahmad et al. indicated that in the context of PTEN loss, PPARγ activation promotes the development of prostate tumors [48]. Data from cBioPortal reveal that the *PPARG* gene is amplified or upregulated in a subset of castration-resistant prostate cancers [48]. PPARγ is also expressed in neuroendocrine prostate cancers [49]. Therefore, increased levels of PPARγ may promote the growth of advanced prostate cancers. Since both GR and PPARγ have been linked to enhanced tumor growth, the increased expression of these nuclear receptors may help prostate cancer cells to overcome metformin-induced reductions in AR.

Our data suggest that growth inhibitory concentrations of metformin also alter expression of p53 target genes. p53 is a well characterized tumor suppressor that regulates cell cycle progression and apoptosis. While p53 mutations have been detected in localized prostate cancer, they are more frequently found in metastatic tumors [50]. I. Ben Sahra et al. initially suggested that the anti-proliferative effect of metformin in LNCaP cells was not linked to activation of AMP kinase but instead required the presence of functional p53 [9, 18]. Additional studies have linked p53 to the ability of drug combinations involving metformin to induce apoptosis in prostate cancer cells [34, 35]. Our data support the idea that p53 function is a key component of the anti-proliferative response to metformin. In C4–2 cells, metformin exposure induced expression of DDIT4/REDD1, which has been linked to the anti-proliferative effects of metformin in LNCaP cells, as well as other p53 target genes that play key roles in cell cycle progression and apoptosis. Furthermore, the C4–2 and 22Rv1 cells, which express wild-type p53, responded better to metformin than the p53 null PC-3 cells [36, 51]. However, PC3 cells also express little to no AR protein. There is evidence of crosstalk between the AR and p53 signaling pathways in prostate cancer. p53 suppresses the expression of AR in LNCaP cells as well as normal human prostate epithelial cells [52]. Conversely, ligand-induced activation of AR has been reported to decrease p53 protein levels in LNCaP cells [53, 54] and decrease expression of p53 target genes within the ventral prostate [53]. Therefore, the ability of metformin to regulate p53 target genes in C4–2 cells and other AR-positive prostate cancer cell lines may result from increases in p53 signaling that occur downstream of metformin-mediated suppression of AR. Also, it is important to note that 43% of the metformin-regulated genes we detected by RNA-seq are not direct targets of either p53 or AR. Our data demonstrate that metformin regulates the expression of genes in additional signaling pathways known to drive the growth of prostate cancers, such as the TGF-β, Wnt, and ErbB/MAPK signaling pathways (reviewed in [55–58]). Therefore alterations in other signaling pathways may contribute to the net anti-proliferative of metformin within p53-positive prostate cancer cells.

To date, multiple studies have examined the combined effects of metformin with other medications in vitro and in vivo. Some groups have explored combinations of metformin and drugs that interfere with AR signaling. The combination of metformin and the antiandrogen bicalutamide synergistically decreased the growth of AR-positive LNCaP cells, LAPC4 cells, and PDX models of prostate cancer [59, 60]. Co-treatment with metformin and either the antiandrogen enzalutamide or androgen synthesis inhibitor abiraterone increased death of AR-positive castration sensitive LNCaP and VCaP cell lines [61]. Other drug combinations involving metformin have also shown to be effective at reducing prostate cancer cell growth. The combination of metformin and vitamin D3, a ligand for the vitamin D receptor, inhibited proliferation of DU145 cells in a synergistic manner [62]. The combination of metformin and taxanes such as paclitaxel and docetaxel reduced proliferation and viability of human prostate cancer cells [63, 64]. D. lliopoulous et al. demonstrated that combined exposure to metformin and the anthracycline doxorubicin suppressed prostate cancer xenograft growth [65]. The combination of metformin and the histone deacetylase inhibitor valproic acid also reduced growth of LNCaP and PC-3 xenografts in nude mice [66]. Furthermore, synergistic decreases in prostate tumor growth were noted following in vivo exposure to metformin and EZH2 inhibitors [67]. The results of our study suggest that additional medications could be combined with metformin to reduce tumor growth. For example, metformin could be paired with drugs that target ErbB or Wnt signaling. Inhibitors of the ErbB signaling pathway are currently used to treat patients with breast cancer, while clinical trials have tested the effectiveness of drugs targeting the Wnt pathway in various solid tumors. In addition, drugs such as APR-246 that re-activate mutant p53 within cancer cells could enhance the overall effectiveness of metformin. Additional studies are required to determine if treatment strategies that involve metformin and drugs that target these pathways would significantly alter prostate tumor growth and progression.

## Conclusions

In summary, metformin regulates activation of the AR signaling pathway as well as other pathways critical for the growth and survival of human prostate cancer cells. The identification of these additional metformin-regulated pathways can be used to design new combination therapies to control the growth of aggressive prostate cancers.

## Supplementary Information

**Additional file 1.**

**Additional file 2.**

## Data Availability

The RNA sequencing dataset generated and analyzed in this publication has been deposited in NCBI’s Gene Expression Omnibus and is accessible through GEO Series accession number GSE196343 [https://www.ncbi.nlm.nih.gov/geo/query/acc.cgi?acc=GSE196343].
