# Supplementary material for: Metformin regulates multiple signaling pathways within castration-resistant human prostate cancer cells
Source: BMC Cancer. 2022 Sep 29;22:1025. doi: 10.1186/s12885-022-10115-3 (PMC9520831; doi:10.1186/s12885-022-10115-3)

**Supplementary Figure 1: Complete blots for data shown in Figure 4A.**
Red boxes denote areas of the blots included in the figure.

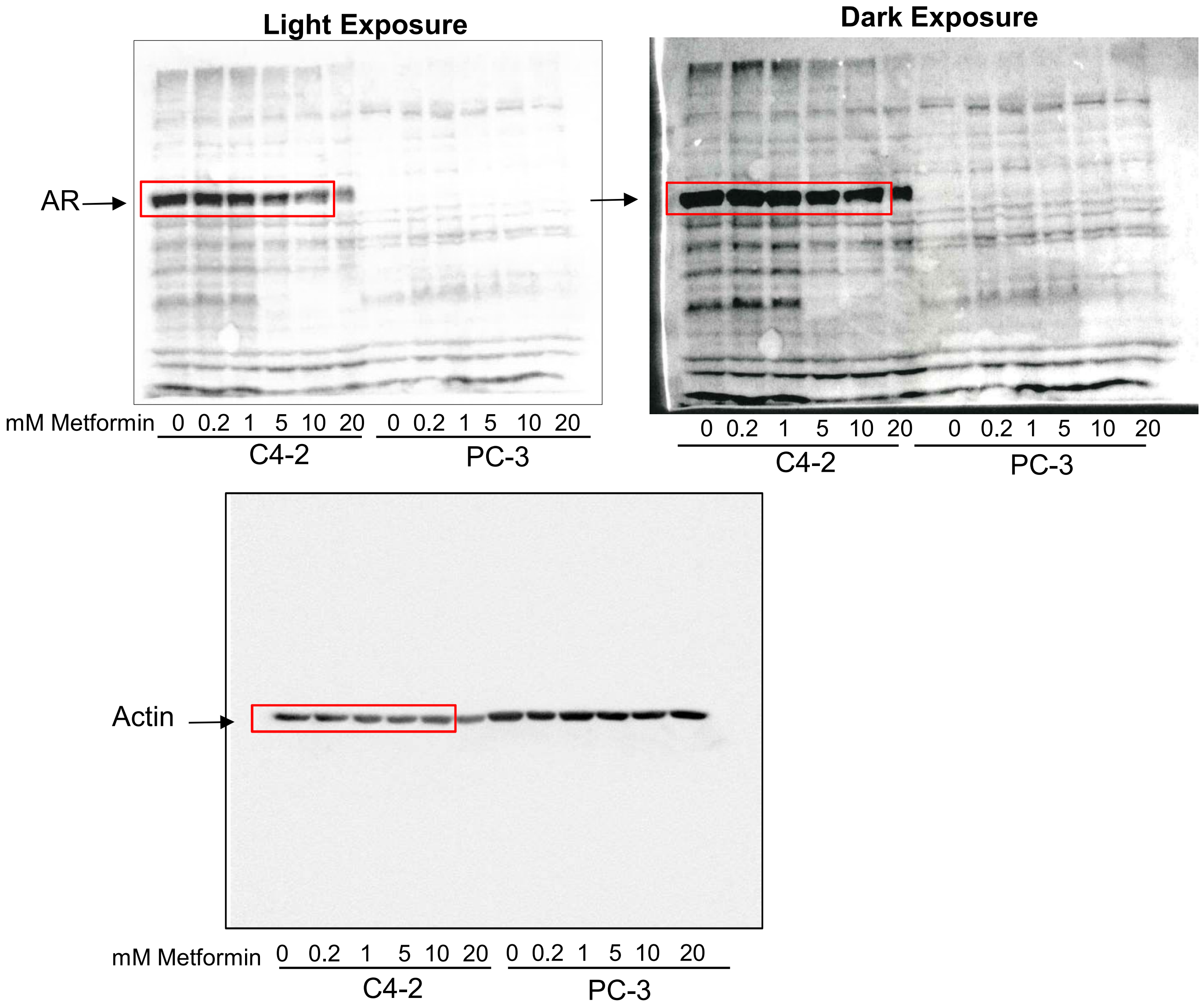

Supplement: Supplementary file 1 — Additional file 1. [file 12885_2022_10115_MOESM1_ESM.pdf]
